# Should I stay or should I go? The effect of London’s terrorist attack on the educational choices of Muslims

**DOI:** 10.1007/s13209-023-00282-2

**Published:** 2023-06-09

**Authors:** Diego Astorga-Rojas

**Affiliations:** Pontificia Universidad Javeriana, Cra. 7 No. 40 - 62, Bogotá, Colombia

**Keywords:** Education, Terrorism, I20, I29, J15

## Abstract

This paper evaluates how the July 2005 London terrorist attacks affected Muslim teenagers’ education plans and decisions. The attacks triggered a violent backslash against the Muslim community, which could have affected their incentives to continue in full-time education. I examine panel data on educational attitudes from the “Next Steps” Survey in England and use the month the survey was administered to divide individuals into treatment and control groups. I find that the attacks negatively affected the education plans of Muslims, but not those of any other major religious group. The probability of planning to continue in non-compulsory full-time education decreased by around 4.4% points for Muslims after the attacks. This corresponds to a 69% increase in individuals who were not sure whether to continue or drop out of full-time education. However, this change in plans appears to be a temporary reaction, since it did not affect students’ actual decisions two years later.

## Introduction

Identity—a person’s sense of self—is a key factor in individuals’ economic decisions. Identifying with a group can change the individual payoffs of a person’s actions as well as those of others. Akerlof and Kranton (2000) incorporate this reasoning into a simple economic model and arrive at different conclusions from the standard case. When studying poverty, they find that social exclusion can cause agents to avoid remunerative activities under a model in which identity plays a role.

This paper investigates the interaction between identity and education. It studies how individuals’ education plans are influenced by a change in the environment faced by the community or group with which they are associated, using the case of Muslim teenagers in England before and after the London terrorist attacks of July 7, 2005.

The London bombings (called 7/7) were the country’s first ever suicide attack. Four British Islamist men detonated four bombs in the center of London, killing 52 civilians and injuring over 700 more. The media reported a violent backslash against the Muslim community across the UK in response to these attacks.^1^ Kielinger and Paterson (2013) and Hanes and Machin (2014) found significant increases in hate crimes against Asians and Arabs in the UK almost immediately after the terror attacks of 9/11 and 7/7. These crimes subsequently decayed, but remained at higher than pre-attack levels for months afterward.

I use data from the ’Next Steps’ survey on English teenagers’ educational attitudes to understand whether (and how) the tense environment generated by the attacks changed Muslim teenagers’ education plans and decisions. My analysis proceeds in four steps.

First, I document whether the respondents who identified themselves as Muslims reported that they planned to continue in full-time education after the age of 16. I rely on the exogenous timing of the attack to divide respondents between the treatment group (those interviewed in 2005 after the bombings) and control group (those interviewed in 2005 before the attack). As the groups differ in some important characteristics such as family composition and work status of the main parent, I exploit the panel dimension of the data to study individual-level changes in education plans between 2004 (when the first wave of the survey was administered) using a fixed effects approach. I also use a differences-in-differences approach as an alternative methodology.

I find that the terrorist attack had a robust negative effect on Muslim students’ education plans. The average marginal effects suggest that the probability of planning to continue in full-time education decreased by around 4.4 percentage points for these respondents. This corresponds to a 69% increase in individuals who *were not sure* whether to continue or drop out of full-time education.

In a second step, I repeat the same analysis for different religions as a placebo test and find that the terrorist attack had no effect on the education plans of individuals who identified themselves as Christians, Hindus or atheist. There was, however, a negative effect for Sikhs. This community was also negatively affected as reported in the news, mainly because of ignorance of the perpetrators and the attention drawn by the turbans they wear.^2^ In a second placebo test, I assume the attack was a month earlier and find no effect on Muslim teens’ education plans.

Then, by comparing Muslims with other religions, I find suggestive evidence of a decrease in well-being and more expectations of discrimination in the future job market after the attacks for the Muslim group. Although these results are not novel (see Hole and Ratcliffe 2020), they serve as possible mechanism for the change in education plans because of the attacks.

Third, I find that the effects are similar in magnitude for females and males, although only the latter is statistically significant. I also find a significant effect for both Muslims who were born in the UK and those who were not, yet the effects seem larger for the latter. The effect is also larger for the Muslims living in London. This is consistent with Hanes and Machin (2014) that find a initial larger impact on hate crimes (such as name calling or treats to their Mosques) in London compared to other regions. In other words, results seem to point that the negative reaction in education plans might be driven by the Muslims that experienced a worst backslash against their community and that were less integrated in the host society, as often is the case with migrants.

On top of that, I find that the uncertainly about education plans among Muslim teenagers in the wake of a negative event is primarily driven by two subgroups: those with parents who have basic or no education, and those whose parents were not working. Interestingly, I found no effect for Muslims with working parents or parents with intermediate or higher education levels.

Finally, I follow the respondents over time to determine what their main activity is at age 17 (after education is no longer compulsory). I find that the answers regarding education plans from 2004 and 2005 do *not* help predict whether education or apprenticeship is the respondent’s main activity in the Muslim sample, but they do so for the rest of the respondents. Furthermore, using information from the 2001 and 2011 censuses supplied by the Office of National Statistics, I analyze the change in educational attainment by religion for cohorts that were close to age 16 (when English students decide whether to remain in formal education) in 2005. The percentage of Muslims aged 19–21 with high qualifications (level 4 or more) increased from 2001 to 2011. This increase was greater than for many other religions, which suggests the initial negative reaction toward continuing in full-time education cooled down and faded as people had more time to think about their choices. This pattern is also consistent with Zorlu and Frijters (2019), who find that the happiness of Muslim migrants in Europe initially fell after the 9/11 attacks but caught up in subsequent years.

### Related literature

Terrorism can significantly affect aggregate economic outcomes such as GDP (Abadie and Gardeazabal 2003), investment (Eckstein and Tsiddon 2004; Abadie and Gardeazabal 2008), stock prices (Berrebi and Klor 2010; Straetmans et al. 2008), crime (Draca et al. 2011) and tourism (Sandler et al. 1992). Recent studies have focused on how it can influence individual outcomes such as happiness and well-being. For example, Metcalfe et al. (2011) finds that self-reported well-being decreased in the UK after the attacks of September 11, 2001. Likewise, Ahern (2018) provides evidence that terrorism has a detrimental effect on individual psychological traits: it decreases trust and subjective well-being, as well as respondents’ opinions of the importance of creativity and freedom. Similarly, Coupe (2017) document a decrease in optimism in France caused by the attacks in November 2015 in Paris. Clark et al. (2020) add to this line of research by studying *experienced* (rather than *subjective*) well-being. They document a decrease in happiness and an increase in negative emotions that lasted for at least one week in the USA after the Boston marathon bombing of 2013.

More closely related to the current study, the differential effects of terrorism on minorities have also been studied for outcomes beyond education. Kaestner et al. (2007) find that the September 11th attacks did not affect the employment or working hours of Arab or Muslim men in the USA, although they were associated with a 14–16% decline in their real weekly earnings; Cornelissen and Jirjahn (2012) reports a negative effect in earnings for Arabs in Germany after the September 11th attacks. Regarding the London’s attcks, Braakmann (2007) finds no impact on the number of hours worked, real wages or employment probabilities in London for Arab or Muslim men. However, Rabby and Rodgers III (2010) reports that London’s metro attacks on 7/7 negatively affected the labor outcomes of Muslims aged 16–25.

Prior research has also assessed how Islamist terrorist attacks influence the social environment toward Muslims—and how the Muslim community’s attitudes and actions respond to such changes. Hanes and Machin (2014) verifies that there was a significant increase in hate crimes against Asians and Arabs in the UK almost immediately after the terror attacks of 9/11 and 7/7. These crimes subsequently decayed, but remained at higher than pre-attack levels a year later. Elsayed and de Grip (2018) shows that Dutch survey respondents’ perceptions of immigrants’ integration decreased after the terrorist attacks in London for Muslim (but not non-Muslim) immigrants. Both papers provide support for the idea that the attacks increased the perceived level of discrimination or segregation against the Muslim community.

Lauderdale (2006) finds that 6 months after 9/11, Arabic-named women in California experienced a moderate increased in the risk of low birth weight compared to similar women who gave birth the year before. Hole and Ratcliffe (2020), focusing on subjective well-being, demonstrate a decrease in self-reported happiness for Muslim teenagers in the UK after the London bombings, especially among girls. They also find an increase in expected discrimination in the job market because of their religion. Zorlu and Frijters (2019) find a decline, and then a subsequent return to average happiness, among the general Muslim migrant population relative to others after 9/11. They also document a persistent decline in happiness for Muslim migrants from the Middle East, which highlights the potential influence of this type of attack on migrants’ integration into their host society. Romanov et al. (2012) studies the same question but in the Israel–Palestine context. They find that Palestinian terrorism has no effect on the happiness of Jewish Israelis and a negative, but not persistent, effect for more than one day on Arab citizens. They attribute Arabs’ initial negative reaction to increasing concerns that they will be discriminated against.

This paper advances this line of research by focusing on the plans and decisions of Muslim teenagers related to acquiring human capital in reaction to the environment provoked by terrorist attacks. Much of the literature that analyzes how terrorism affects well-being suggests that depressed adolescents might decide to study less, as one of the main arguments of why should we care (see, for example, Hole and Ratcliffe (2020)). However, this channel has not been directly studied.

Most previous research that has explored the differential effect of terrorism on the outcomes of the minority identified with the perpetrators vs. the rest of the population has used different races or religions as a control group in a difference-in-differences setting. The results of these papers rely on the parallel-trends assumption for both groups in the absence of a treatment. Although I also use a similar difference-in-differences approach for the first set of outcomes, the timing of the interviews allows me to establish a control group *within* the same religion and to use other religions as placebos, making the parallel assumption less of a concern. The panel structure of the data also lets me use a fixed effects approach, which is often difficult to do in these kinds of studies, which further alleviates concerns about parallel trends.

More related to education outcomes and violence, Brück et al. (2019) study the effect of the Israeli–Palestinian conflict on various education outcomes for Palestinian high school students in the West Bank during the Second Intifada. They find that the conflict negatively affected their grades and made them less likely to attend university in the future. The authors identified two possible main channels for this result: (1) the conflict-induced deterioration of school infrastructures and (2) the worsening of the students’ psychological well-being due to direct exposure to violent events. However, this type of violence could have very different consequences from those caused by terrorist attacks. The attacks during the Intifada were more regular and affected the supply of education via infrastructure, whereas terrorist attacks are infrequent and did not affect the school’s infrastructure.^3^

Finally, the paper suggests two channels through which terrorism can affect education plans. These channels have been extensively discussed in the literature: (1) the effect on mental health and well-being and (2) the effect on job market expectations.

As explained before, several papers have reported a decline in well-being (Hole and Ratcliffe 2020) or happiness (Zorlu and Frijters 2019; Romanov et al. 2012) following terrorist attacks. This phenomenon is linked to a body of literature that examines the impact of stress, depression, and mental health on educational outcomes. For instance, Fletcher (2008) found a correlation between depression and educational attainment, particularly among females, across various dimensions, such as high school dropout rates, college enrollment and the type of college attended. Similarly, Johnston et al. (2014) found similar results, focusing on educational progress, even after accounting for measurement error and sub-reporting in the assessment of individuals’ mental states. By using specific genetic markers as a source of exogenous variation in depressive symptoms, Ding et al. (2009) found evidence suggesting poorer academic performance.

The literature on expectations in the job market and human capital accumulation is more divided about their conclusions. Coate and Lourie (1993) argue that if workers expect to face bias in the hiring process, their incentives to invest in job relevant skills (such as education) are reduced, inducing a self-fulfilling prophecy of difference in skills across groups. Lundberg and Startz (1983) present a model where unobserved human capital investments, combined with less informative productivity signals for one group reduce the pay and incentives for ethnic minorities to acquire skills in a model of statistical discrimination. Lang and Manove (2011) argues that in fact the opposite relation between discrimination expectations and education could exist. Given that schooling is an investment in human capital that can be seen, it can be used as a signal for higher ability and productivity, such that minorities can use it to eliminate the statistical discrimination. In line with this line of thought, Arcidiacono et al. (2010) finds that college plays a much more direct role in revealing ability to the labor market than high school, explaining the wage gap face by blacks in high school market that is not found in the college market. They argue that this result can explain why in the fact documented by the literature that, conditional on ability, blacks are more likely to get earn a college degree.

In the case of Bennett et al. (2015), they provide a model where the decision to invest in education depends on the productivity level of the migrants (high and low productivity) and whether negative actions are targeted toward both groups or only one of them. They explain the educational gap between migrants and natives by proposing a Becker-style taste discrimination model within a search and wage bargaining setting in which agents have an educational choice. In their model, if negative attitudes toward high- and low-productive immigrants increase, immigrants’ skill level will decrease because of the worst labor outcome perspectives. When only low-productivity workers face negative attitudes, however, immigrants’ education level can increase. They find that in regions in Denmark that have more negative attitudes toward immigrants, immigrants are more likely to stay in high school.

The paper is related to both mechanisms. Like Hole and Ratcliffe (2020), I found that the London terrorist attacks had a negative impact on the well-being of Muslims compared to individuals from other religions. Additionally, I documented an increase in expectations of discrimination in the job market for the Muslims. The results of the study suggest that this decrease in well-being and expectation of discrimination made the returns of education more uncertain for the Muslim community, thereby making their decision to continue in full-time education more unclear.

The rest of the chapter is structured as follows. The next section explains the ’Next Steps’ survey in more detail. Section 3 describes the empirical strategy and main specifications. Section 4 presents the results for the education plans and the heterogeneity across sub–populations. Section 5 analyzes the students’ education decisions, and Sect. 6 concludes.

## Data

The data on young Muslims is taken from The Longitudinal Study of Young People in England (LSYPE), also known as Next Steps.^4^ It is a major panel study of young people containing information on the teenagers and their parents about educational attitudes and family backgrounds.

The study began in 2004, when most of the sample were aged 13–14. The study over-sampled deprived schools and minority ethnic groups, to “ensured that within a deprivation stratum, all pupils within an ethnic group had an equal chance of selection” Department for Education (2011). According to the 2011 Census, 46% of Muslims lived in the 10% most deprived local authority districts in England (Ali 2015), so the panel’s construction helps generate a more representative sample of the country’s Muslim community.

I focus on respondents who defined themselves as Muslims. I define education plans as a dummy indicating whether the teenager plans to stay in full-time education based on their answer to the following question in the 2004 and 2005 waves of the survey:

When you are 16 and have finished Year 11 at school what do you want to do next...

- ...stay on in full-time education, either at the school you are at now or somewhere else
- ...or leave full-time education
- ...leave full-time education but return later (e.g., Gap Year) SPONTANEOUS ONLY
- Don’t know

Year 11 is the last year of compulsory education in England. I code the answers “don’t know” and “leave full-time education” as 0 for the variable *Education Plans*. I assign a value of 1 if the respondent said she was returning to full-time education or planning to take a gap year before returning to full-time education. In fact, as “Appendix A.2” in Tables 10 and 11 show, the results come from more Muslims stating they “don’t know” whether to continue in full-time education after the attack, rather than answering their plan to leave full-time education. For this reason, this variable should be interpreted as one that indicates the individual’s certainty about his intention to continue in full-time education after finishing the compulsory years.^5^

In addition to their education plans (whether they plan to continue in full-time education after the age of 16), I observed whether they were born in the UK, the education of the main parent and working status.^6^ I also focus on household type (whether the teenager lives in a married/cohabiting household or with a single parent).

Table 1 reports the mean of the variables at baseline for the main sample used in the paper. It is divided between those interviewed after the terrorist attack (treatment group) and those interviewed before (control group).

**Table 1:** Descriptive statistics in 2004: main sample of Muslims

	Treatment group	Control group	Difference
*A. General information*			
Education Plans	0.956	0.931	0.025
Born in the UK	0.832	0.759	0.073**
*B. Working status-main parent*			
Working Full Time	0.259	0.204	0.055**
Working Part Time	0.097	0.090	0.007
Not Working	0.644	0.706	$-$0.062**
*C. Education level-main parent*			
No qualifications	0.632	0.650	$-$0.018
Basic	0.050	0.058	$-$0.008
Intermediate	0.215	0.177	0.038
Advance	0.103	0.115	$-$0.012
*D. Household type*			
Single parent	0.168	0.225	$-$0.057**
Married or cohabiting	0.832	0.775	0.057**
Observations	340	844	

The treatment group comprises Muslim respondents interviewed after the terrorist attack (August–September 2005), and the control group contains Muslims interviewed before the attack (April–June 2005). *Education Plans* is a dummy indicating whether the teenager plans to stay in full-time education (either continuously or after a gap year)

***p < 0.01, **p < 0.05, *p < 0.1

The treatment group has a lower proportion of individuals who were not born in the UK. It also has a larger share of teenagers with a parent working full time and who live in a married or cohabiting household.

“Appendix Table 9” reports the same variables for Muslim vs. non-Muslim respondents. On average, the former teenagers are more certain about their plans to continue in full-time education than the latter. They differ in all other characteristics from the rest of the population. Fewer Muslims were born in the UK than their non-Muslim counterparts, the education of the main parent is lower for the Muslim respondents, and more of them are unemployed. These differences demonstrate that using the entire population as a control group for the Muslim community is a hard assumption to make. Although this problem could be addressed by using a more similar sub-population, the fact that the analysis involves individuals of the same religion represents an improvement upon similar prior studies.

**Fig. 1:**
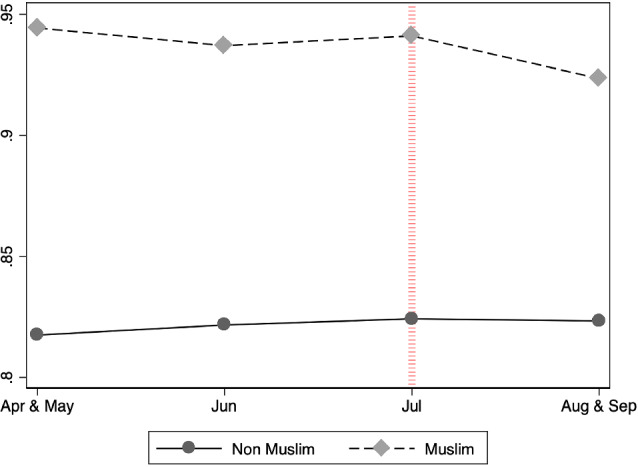
Proportion of people with education plans = 1 by religion and month of interview in 2005. *Notes:* *Education Plans* equals 1 if the student expressed the intention to continue in full-time education at the age of 16, and 0 otherwise. The solid (dashed) line indicates the proportion of non-Muslims (non-Muslims) in each month with education plans equal to 1. The red line denotes the date of the terrorist attack. The months of April and September are stacked with May and August, respectively, because few Muslims were interviewed in April $(n = 26)$ and September $(n = 65)$

Figure 1 displays the proportion of teenagers each month in 2005 who reported that they planned to continue in full-time education. After the attack (denoted by the red vertical line), fewer Muslim respondents reported planning to continue in full-time education; there was no change for non-Muslim respondents. “Appendix” Figs. 5 and 6 present the distribution of Muslim interviewees according to their education plans in 2004 and 2005 by month of interview in 2005. They are the same graph but 6 is zoomed in so the changes can be appreciated. Figure 7 shows the same distribution (only zoomed in) for the non-Muslim interviewees. Together, these graphs tell a similar story as Fig. 1.

**Fig. 2:**
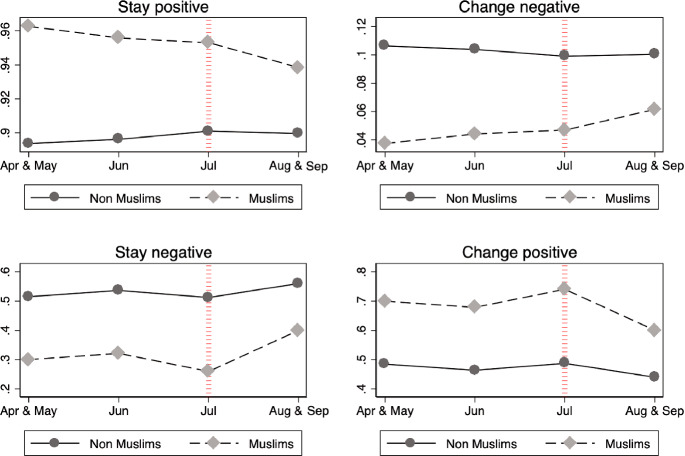
Distribution of change in education plans by month of interview in 2005-conditional on the education plans in 2004. *Notes* The upper panel displays the distribution of the education plans of Muslims (dashed line) and non-Muslims (solid line) in 2005 by month, among those who expressed their intention the previous year to remain in full-time education at the age of 16. The upper-left graph shows the proportion of people that maintained the same idea in 2005 (Stay positive) and the upper-right figure displays the proportion of people who in 2005 were unsure about continuing in full-time education (Change negative). The bottom panel performs the same analysis on those who expressed in 2004 that they were unsure about remaining in full-time education at the age of 16. Change negative =1 if they remained unsure in 2005 and Change positive =1 if in 2005 they expressed an intention to continue in full-time education. The red line in July denotes the date of the terrorist attack. The months of April and September are stacked with May and August, respectively, because few Muslims were interviewed in April $(n = 26)$ and September $(n = 65)$

Figure 1 demonstrates that after the attack, more Muslims expressed that they were unsure about continuing in full-time education. Figure 2 conditions on their education plans as indicated in the 2004 survey to study whether this increase in uncertainty comes from (1) people who had planned in 2004 to continue studying but changed their intention after the attack (Change negative) or (2) an increase in the number of people who expressed in both years that they were unsure about whether they would continue to study (Stay negative).

Conditional on their answers in 2004, more Muslim students negatively changed their education plans (in 2004 they planned to continue in full-time education but in 2005 were no longer sure); more Muslim students also expressed being unsure of their education plans in both 2004 and 2005. The analysis yields no clear trend for non-Muslim students after the attack. While these graphs present suggestive evidence that the London bombings in July 2005 might have negatively influenced Muslim respondents’ education plans, I turn to the regression analysis to quantitatively estimate this effect.

## Empirical strategy

### Changes in education plans

To determine whether the attack caused Muslim students to change their education plans, I divided the Muslim respondents into treatment and control groups based on the date of their interview in 2005 (those interviewed in August 2005 or later were placed in the treatment group). The terrorist attack is assumed to be unexpected to avoid endogeneity concerns.

**Fig. 3:**
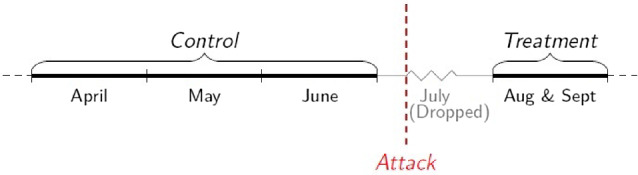
Treatment and control groups by month of interview in 2005

The interview date determines the individual’s exposure to the shock introduced by the treatment (bombings). An individual interviewed after the attacks has more information to internalize (such as the new environment toward his community) when completing the survey and discussing his future education plans (Fig. 3).

Individuals in the treatment group would be treated only in 2005; 2004 serves as the baseline survey. I drop individuals surveyed in July 2005 from the analysis because the data only contains the month of the interview; therefore, I do not know if they were surveyed before or after the attack. The main results are robust to including this month in the treatment group.

Since the control and treatment groups differ in certain baseline characteristics, I use a panel fixed effect approach to identify the effect of the terrorist attack on Muslim respondents’ education plans.

1 $$\begin{aligned} Y_{it} =&\beta _{1}Year_{2005} + {\beta _{2}}Treated_i*Year_{2005} + X_{i,2004}'*t*\gamma + \lambda _i +\nu _{it} \end{aligned}$$

Equation (1) is the main specification for the change in education plans. $$Y_{it}$$ is a binary indicator that takes a value of 1 if individual *i* in year *t* plans to continue in full-time education after age 16. *Treated* is a dummy indicating assignment to the treatment group, $$Year_{2005}$$ is a dummy coded 1 if the individual was interviewed in 2005, and $$X_{i,2004}$$ is a vector of individual characteristics at baseline that could affect their plans to keep studying. The controls used are the same as those presented in the descriptive statistics in Table 1. The person fixed effect ($$\lambda _i$$) captures all time-invariant unobservables. The baseline characteristics $$ X_{i, 2004}$$ are interacted with a time trend.

The coefficient of interest is the interaction coefficient $\beta _2$, which captures the effect of answering after the attack. Following Abadie et al. (2022), I cluster the standard errors at the student level, but this makes little difference, as the significance remain the same if I cluster at the school level (the primary sample unit). I also use a difference-in-differences methodology in both a linear probability model and a probit model as alternative estimations.

2 $$\begin{aligned} Y_{it}&= \alpha _0+ \alpha _{1}Year_{2005} + \alpha _{2}Treated_i + {\alpha _{3}}Treated_i*Year_{2005} \nonumber \\&\quad + X_{i,2004}'*\gamma + u_{it} \end{aligned}$$

In Eq. (2), the effect of the terrorist attack on education plans would be identified by $\alpha _{3}$, either in the case of the probit model or the linear probability model.

Finally, I allow Eq. (1) to be estimated in a more flexible manner by studying the changes to education plans by month of interview in 2005, instead of just a dummy of being interviewed months after July 2005 (treated) or months before (control).

3 $$\begin{aligned} Y_{it}&= \beta _{1}Year_{2005} + {\beta _{2}}May_{i}*Year_{2005} + {\beta _{3}}August_{i}*Year_{2005} \nonumber \\&\quad + X_{i,2004}'*t*\gamma + \lambda _i +\nu _{it} \end{aligned}$$

From this specification, $\beta _1$ indicates the change in education plans between 2004 and 2005 of those students who were interviewed in June 2005 (reference group), $\beta _{2}$ captures the difference in the change of education plans between those interviewed in June and those interviewed in May or April, while $\beta _{3}$ captures the same difference between the reference group and those interviewed in August or September. If the change in education plans is due to the terrorist attacks, one should expect $\beta _{2}$ to be non-different from zero while $\beta _{3}$ should be statistically different from it.

A potential concern is that the news that London would host the 2012 Olympics (this was announced one day before the attacks) could translate into better expectations in terms of labor opportunities in different sectors. This would increase the outside option of continuing in full-time education, making teenagers more likely to change their education plans and drop out of full-time education. Another concern is that an unobserved fixed characteristic that is correlated with both education plans and the treatment status (month of interview in 2005) could be driving the results. To address these concerns, I propose two placebos.

#### Effect on other religions

The argument is that after the terrorist attack, the level of violence targeted at the Muslim community increased, so the most affected students were those who identified themselves as Muslims. I therefore estimate Eq. (1) separately for Hindus, Christians, Sikhs and atheists. The effect of the attack should be economically lower and statistically insignificant if the paper’s argument is true. And if the Olympics affected teenagers’ education plans, Muslims were unlikely to be affected differently than other religions.

#### False treatment—acting like the attack was in June 2005

I also conduct a second placebo test within the Muslim population. From the control group, I create a false treatment group (those who were interviewed in June 2005) and rerun the analysis in Eq. (1). Intuitively, if the effect is driven by the actions after 7/7, there should be no differential effect in this regression between those interviewed in June vs. those interviewed in April and May, as both groups are reporting their education plans in a similar environment.

### Educational decisions

I start by creating a dummy that takes a value of 1 if the individual is in full-time education at age 17. To construct this variable, I used individuals’ responses about their main activity (education, employment, apprenticeship or inactive). To avoid seasonality, the survey specifies that even if the individual is working in the summer, he should respond “education” if that is his main activity in the rest of the year.

The difference-in-differences strategy used to determine change in education plans cannot be used to assess whether Muslim teenagers followed these plans for two reasons. First, the information about the main economic activity is cross-sectional, so the time dimension of the panel is lost. Second, even though the treatment and control groups were defined based on the timing of their interview in relation to the terrorist attack, the control group could still have been affected *after* the attack. In other words, the Muslims interviewed before the attack were also exposed to the backlash against their community because of the attacks, so they are no longer a good control group.

To analyze whether the intention to stay in full-time education mattered, I estimate a probit based on the change in plans between 2004 and 2005:

4 $$\begin{aligned} \begin{array}{ll} Edu_{i}=&{}1(\alpha _0 + \alpha _{1}Change\,negative_i + \alpha _{2}Change\,positive_i \\ &{}+ \alpha _{3}Stay\,positive_i + X_{i}'\gamma _2 + \epsilon _{i}\ge 0) \end{array} \end{aligned}$$

In Eq. (4), the dependent variable $Edu_{i}$ is a dummy indicating whether the respondent’s main activity at age 17 is education. $Change\,negative$ takes a value of 1 if initially (in 2004) the person was planning to stay in full-time education but changed her mind in 2005. Similarly, $Stay\,positive$ and $Change\,positive$ take a value of 1 if she always planned to stay in full-time education or if she was unsure of staying in full-time education and the next year she changed her mind, respectively. $Stay\,negative$ takes a value of 1 if the education plans in both years were unsure of staying in full-time education; these respondents were left out as the referenced group in Eq. (4).

This analysis compares whether the responses related to education plans have the power to predict the individual’s actual decisions, for both Muslims and non-Muslims. Although this analysis cannot claim causality, it suggests the persistence of individual responses in their eventual decisions.

## Changes in education plans

Columns 1–3 of Table 2 report the estimates of Eq. (1). Columns 4–7 present the results of Eq. (2) in a linear probability model and also a probit. The results are significant and robust to the controls in each case. Only individuals who did not move schools and whose region was not missing were included in the sample for Columns 3, 5 and 7. This was done to prevent any bias resulting from changes in educational resources or environment.^7^

**Table 2:** Effects of the terrorist attack on education plans

	Dependent variable: education plans						
	FE			LPM		Probit	
	(1)	(2)	(3)	(4)	(5)	(6)	(7)
Treated				0.026*	0.029**	0.229	0.260*
				(0.014)	(0.014)	(0.140)	(0.143)
$Year_{2005}$	0.009	0.041	0.042	0.009	0.011	0.080	0.092
	(0.010)	(0.032)	(0.033)	(0.010)	(0.010)	(0.082)	(0.083)
Treated*$Year_{2005}$	$-$0.042**	$-$0.044**	$-$0.049**	$-$0.042**	$-$0.047**	$-$0.359**	$-$0.404**
	(0.019)	(0.019)	(0.019)	(0.019)	(0.019)	(0.163)	(0.164)
Controls	N	N	N	Y	Y	Y	Y
Controls*time trend	N	Y	Y	N	N	N	N
Average Marginal Effects							
						$-$0.039**	$-$0.045**
						(0.018)	(0.018)
Mean of dep. variable	0.937	0.937	0.937	0.937	0.937	0.937	0.937
Observations	2368	2368	2316	2368	2316	2368	2316
R-squared	0.004	0.012	0.012	0.015	0.016	0.039	0.040
Clusters	1184	1184	1158	1184	1158	1184	1158

*Education Plans* is a dummy coded 1 if the respondent planned to continue in full-time education by year 11, regardless of whether they intended to take a gap year. Columns 3, 5 and 7 restrict the sample to non-movers. Controls are baseline characteristics in 2004 presented in Table 1: dummies for working status and education level of the main parent, single or cohabiting household, gender and if the individual was born in the UK. Standard errors are clustered at the individual level

***p < 0.01, **p < 0.05, *p < 0.1

Our preferred results (reported in Column 2) suggest that Muslim teenagers surveyed after the attack were 4.4 percentage points less likely than those surveyed before, on average, to report that they were planning to continue in full-time education. Since the unconditional mean of the education plans of Muslims in 2004 is 0.937 (only 6.3% of Muslims did not plan to continue studying in 2004), a 4.4-percentage-point decrease implies a large increase (69.8%) in the number of individuals who planned to drop out after age 16 or were not sure whether they would continue in full-time education. The results of the linear probability model and average marginal effects found in the probit specification are similar (3.9-$-$4.7 percentage points).

**Fig. 4:**
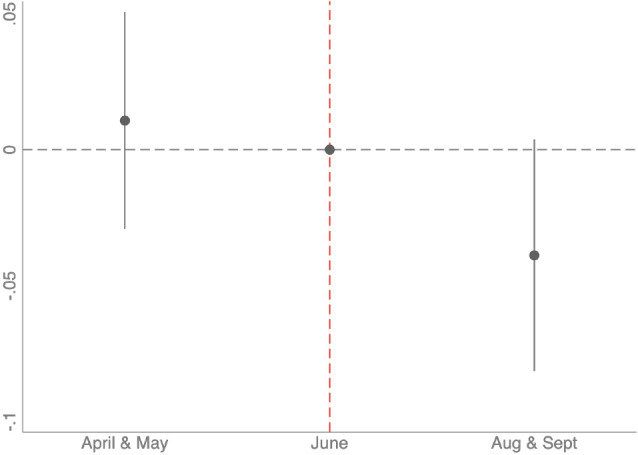
Fixed effect model by month of interviewed in 2005. *Note* Point estimates of $\beta _{2}$ and $\beta _{3}$ in Eq. (3) with their 95% confidence intervals. Controls used for the estimation are baseline characteristics in 2004 presented in Table 1: dummies for working status and education level of the main parent, single or cohabiting household, gender and if the individual was born in the UK, all of them interacted with a time trend. Standard errors are clustered at the individual level

**Fig. 5:**
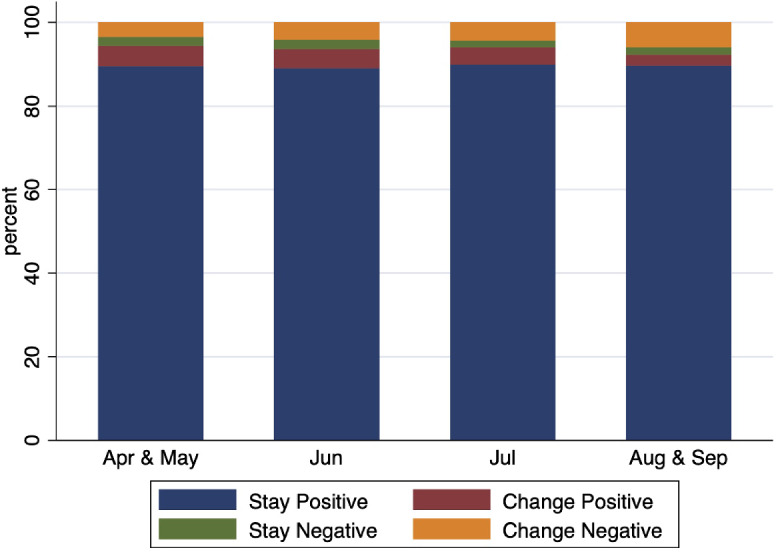
Distribution of change in education plans by month of interview in 2005-Muslims

**Fig. 6:**
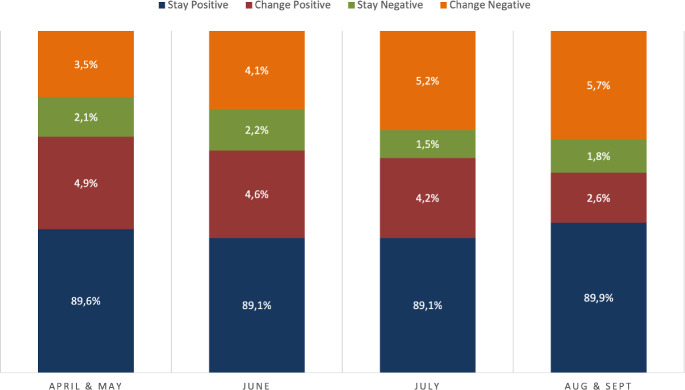
Distribution of change in education plans by month of interview in 2005-Muslims (ZOOM)

**Fig. 7:**
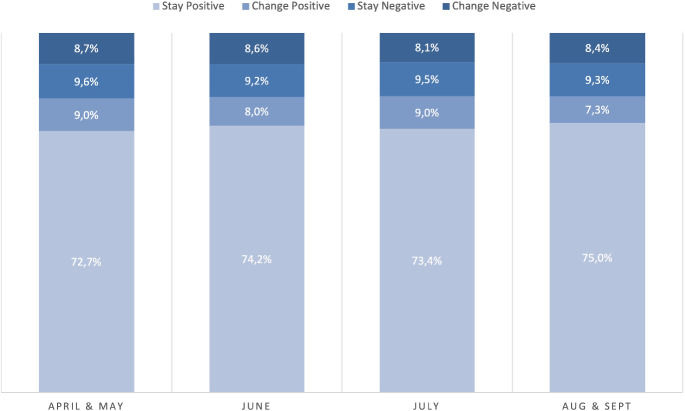
Distribution of change in education plans by month of interview 2005 non-Muslims (zoom)

Figure  4 plots the coefficients of interaction between the month of interview in 2005 and the year 2005 with their 95% confidence intervals, as stated in Eq. (3). The reference group are those Muslims interviewed in June 2005. As expected, there is no significant difference in the changes in education plans between those interviewed in June and those interviewed in April or May. However, being interviewed in the months of August or September in 2005, decrease the probability of planning to continue in full-time education by an average of 3.9 percentage points compared to the group that was interviewed in June 2005. This difference not only remains close to the main effect estimated in Table 2, it also remains statistically different from 0 at the 10% level of significance. A more detailed version of the estimates can be found in Table 12 from the “Appendix.”

### Robustness checks

The suggested explanation for the negative change in education plans for Muslims is that after the attacks, the Muslim community suffered a change in environment that no other group experienced. Thus, estimating Eq. (1) for non-Muslim groups should indicate a zero effect of the terrorist attack. Nor should the timing of the interview have any effect aside from the events in July. Therefore, the change in plans for the Muslims interviewed in April and May vs. those interviewed in June should not differ significantly. Table 3 presents the evidence for the two placebos. The results are insignificant for both, which reassures us that the effect is mainly for Muslim teenagers who were interviewed after the terrorist attack.

**Table 3:** Placebo test

	Dependent variable: education plans			
	FE	LPM	Probit	Observations
	(1)	(2)	(3)	
No religion	$-$0.031	$-$0.032	$-$0.112	5844
	(0.021)	(0.021)	(0.074)	
Christian	0.004	0.005	0.025	8760
	(0.014)	(0.014)	(0.065)	
Hindu	0.004	$-$0.002	$-$0.484	574
	(0.028)	(0.030)	(0.497)	
Sikh	$-$0.068*	$-$0.072*	$-$0.525*	496
	(0.041)	(0.042)	(0.278)	
Other	0.115	0.127	0.594	210
	(0.117)	(0.122)	(0.623)	
False-Treatment	$-$0.010	$-$0.009	$-$0.072	1688
	(0.020)	(0.020)	(0.165)	
Controls	N	Y	Y	
Controls*time trend	Y	N	N	

The coefficients presented represent the interaction between treated and $Year_{2005}$. *Education Plans* is a dummy coded 1 if the respondent planned to continue in full-time education after year 11, regardless of whether they intended to take a gap year. Controls are the baseline characteristics in 2004 presented in Table 1: dummies for working status and education level of the main parent, single or cohabiting household, gender and if the individual was born in the UK. Standard errors are clustered at the individual level

***p < 0.01, **p < 0.05, *p < 0.1

Not only are the results statistically insignificant for non-Muslim respondents; the effect is even of a different sign than the one calculated for the Muslim community. However, there seems to be a negative and slightly significant effect for Sikhs in all specifications, which could be explained by religious ignorance on the part of local (non-Muslim) communities. Several reports of attacks on *gurdwaras* (Sikh temples) were reported after the 7/7 attack. As Sikh spokeswoman Mejindarpal Kaur explained in July 2005, “We are a community that gets targeted because of the way we look and because our people wear turbans.”^8^ If this sentiment was widespread among the Sikh respondents, it could explain why the effect is similar in magnitude and significant at the 10% level.

The false treatment shows that there is no statistical difference between Muslims interviewed in April and May vs. those interview on June. The coefficient in each specification is highly nonsignificant and the magnitude is on the order of four times lower than the main results.

Another potential source of bias is differential attrition of Muslims before and after the attack. Attrition can be due, in part, to Muslims leaving the country after the attacks. If the more positively selected are more mobile, this would lead to a negative selection of those who stay. This would go in the direction of negative effects in education plans, but it would be due to this selection rather than the attack itself. The analysis in the “Appendix” in Tables 14 and 15 shows that this problem is of little concern in the analysis.

I also reran Eq. (1) for the Muslims while taking into account those interviewed in July 2005. Despite the miss-classification of those interviewed before July 7th as if they were treated, the main effects were still significant, albeit at a lower magnitude. The results are presented in Table 13.^9^

### Possible mechanisms

It is important to understand how a terrorist attack can affect the education plans of Muslims. In this section, I explore possible mechanisms that may explain why this could happen.

On the one hand, it may be related to the new environment that Muslims face after the attack. For instance, Muslim teenagers might become targets of bullying, causing them to consider dropping out of school. Alternatively, if they feel more repressed, they may develop negative attitudes toward school, leading to uncertainty about continuing their education.

To investigate whether bullying increased for the Muslim community in school, I used a survey question asking teenagers to report if they had been bullied in any way over the last 12 months. I used this question to create a dummy variable with a value of 1 if the teenager reported being bullied. I then used this dummy variable to check if the likelihood of being bullied increased after the attack. However, there was a problem of many teenagers refusing to answer the bullying question. To address this issue, I only included in the sample those who answered the question in both years.

To study whether attitudes toward school changed, I used two survey questions. The first question asked teenagers if they agreed with the statement *“I am happy when I am at school”*, while the second question asked how much they agreed with the statement *“School is a waste of time for me”*. I constructed two dummy variables, one for each question, with a value of 1 if they agreed or strongly agreed with the statement in question.

Table 4 presents the results of re-estimating Eq. (1) with these three dummy variables as the dependent variables. As shown in the Table, it appears that there was no significant increase in bullying toward Muslims after the attack. Additionally, attitudes toward school appear to remain constant.

**Table 4:** Possible mechanisms—fixed effects

Dependent variable	Bullied	Happy at school	Waste of time
	(1)	(2)	(3)
$Year_{2005}$	0.003	0.006	0.038
	(0.062)	(0.041)	(0.042)
Treated*$Year\_{2005}$	$-$0.038	$-$0.016	0.016
	(0.034)	(0.024)	(0.026)
Controls*time trend	Y	Y	Y
Mean of dep. variable	0.300	0.898	0.105
Observations	2044	2368	2368
R-squared	0.025	0.008	0.005
Clusters	1022	1184	1184

*Bullied* is a dummy coded 1 if the respondent reported to be bullied in anyway over the last 12 months. *Happy at School* is a dummy coded 1 if the respondent agree with the sentence *“I am happy when I am at school”*. *Waste of Time* is a dummy coded 1 if the respondent agree with the sentence “School is a waste of time for me.” Controls are the baseline characteristics in 2004 presented in Table 1: dummies for working status and education level of the main parent, single or cohabiting household, gender and if the individual was born in the UK. Standard errors are clustered at the individual level

***p < 0.01, **p < 0.05, *p < 0.1

Although attitudes toward school may not be the reason why education plans became more uncertain after the terrorist attacks, two potential channels are discussed. First, even if Muslim students feel as happy at school as before, their general well-being may still be affected. Studies by Fletcher (2008), Ding et al. (2009) and Johnston et al. (2014) suggest that well-being is associated with academic performance and outcomes such as the decision to drop out of school.

Secondly, expectations could also be playing a role, as suggested by the literature on expectations and human capital, such as Coate and Lourie (1993) and Bennett et al. (2015). Worse job market expectations could lead individuals to be more uncertain about the returns of their education.

In fact, these two channels have already been identified by Hole and Ratcliffe (2020). Using the same survey, the authors demonstrate that Muslims experienced a decline in well-being following the attack and expected to face greater challenges in the job market due to their skin color, ethnicity or religion. I follow the approach used by the authors and find similar results. These results could be found in Table 16 in the “Appendix.”

### Heterogeneity analysis

I explore whether the effects found in the main regression differ according to gender or being born in the UK vs. elsewhere. The first dimension is important: Hole and Ratcliffe (2020) provide evidence of a decrease in self-reported happiness for Muslim adolescents after 7/7 in the UK, which could be a mechanism to explain why their education plans changed negatively after the attack. Their results are driven by Muslim teenage boys, so the gender dimension might be important. Zorlu and Frijters (2019) also find a temporal decline in happiness in the US Muslim population after 9/11, yet this decline seems more persistent among Muslim migrants coming from the Middle East. Not being born in the UK and being Muslim might complicate this group’s social integration, potentially making the effects of the terrorist attacks more salient.

**Table 5:** Heterogeneity of the effects—fixed effects

	Dependent variable: education plans						
	Baseline	Male	Female	Born in the UK	Not born in the UK	London	Not London
	(1)	(2)	(3)	(4)	(5)	(6)	(7)
$Year_{2005}$	0.041	0.095*	0.006	0.027	0.060	0.003	0.051
	(0.032)	(0.045)	(0.042)	(0.039)	(0.049)	(0.044)	(0.046)
Treated*$Year_{2005}$	$-$0.044**	$-$0.047*	$-$0.041	$-$0.037*	$-$0.086**	$-$0.048*	$-$0.038
	(0.019)	(0.028)	(0.026)	(0.021)	(0.043)	(0.025)	(0.027)
Controls*time trend	Y	Y	Y	Y	Y	Y	Y
Mean of dep. variable	0.937	0.931	0.943	0.933	0.950	0.961	0.923
Observations	2368	1156	1212	1848	520	888	1480
R-squared	0.012	0.028	0.010	0.007	0.071	0.017	0.009
Clusters	1184	578	606	924	260	444	740

*Education Plans* is a dummy coded 1 if the respondent planned to continue in full-time education after year 11, regardless of whether they intended to take a gap year. Controls are the baseline characteristics in 2004 presented in Table 1: dummies for working status and education level of the main parent, single or cohabiting household, gender and if the individual was born in the UK. Standard errors are clustered at the individual level

***p < 0.01, **p < 0.05, *p < 0.1

Table 5 presents the estimates of Eq. (1) for the baseline population and the different groups individually. The results are similar if a linear probability model or probit model is used. The findings are not in line with those of Hole and Ratcliffe (2020) in the sense that only male estimates were statistically significant (at the 10% level), but there was little difference in final education decisions between males and females. Estimates for both groups vary little in magnitude, and female estimates were very close to significance at the 10% level.

When looking at being born in the UK or not, there is a significant effect on both subsets of the population. However, the attack’s negative effect on education plans appears to be larger for Muslims not born in the UK, although this is only tentative evidence as both estimates have relatively large standard errors. The fact that the attack seems to have larger effects for the Muslims not born in the UK is in line with Zorlu and Frijters (2019), as they report a persistent decrease in happiness for the Muslim population from the Middle East in Europe after 9/11. This decrease in happiness was less persistent for the other Muslim population, implying that integration in the host society is an important determinant of how individuals react to a same shock toward their community. If migrants have a lower integration in their host economies, they might struggle more with the negative backlash toward their community, hence, the larger effects.

The last two columns of Table 5 provide a breakdown of the effect between Muslims living in London versus those living outside of London. Previous research by Hanes and Machin (2014) has shown that the initial impact of hate crimes was higher in London than in other regions following the terrorist attacks. The results in Table 5 suggest that the negative effect on education plans may be driven primarily by Muslims living in London, as the coefficient for this group is the only one that is statistically significant. However, it should be noted that the point estimates for Muslims living outside of London are very similar, so this finding is only suggestive and not conclusive.

The fact that the effect could be higher in London would be consistent with the premise suggested in the article that the more toxic the environment for the Muslim community, the more likely the adolescents from this community might be affected. These results would suggest mechanisms for the change in education plans, as results seem to be driven by Muslims that experienced a worst backslash against their community and that have a lower social cohesion. This points to the mechanism of a decrease in happiness affecting the education plans as suggested by Fletcher (2008), for example, as a possible mechanism.

Finally, I classified Muslims based on two proxies of their socioeconomic status: their main parent’s education level and their main parent’s working status. Table 6 presents the result of these estimations. Muslim teenagers from low socioeconomic backgrounds (i.e., those whose main parent had only basic education or was unemployed) were more uncertain about their educational plans following the terrorist attacks.

**Table 6:** Heterogeneity of the effects—fixed effects

	Dependent variable: education plans				
	Baseline	Educated parent	Basic or No education	Working parent	Non-working parent
	(1)	(2)	(3)	(4)	(5)
$Year_{2005}$	0.041	0.076	0.007	0.011	0.093*
	(0.032)	(0.046)	(0.022)	(0.037)	(0.055)
Treated*$Year_{2005}$	$-$0.044**	0.005	$-$0.067***	$-$0.001	$-$0.068***
	(0.019)	(0.030)	(0.024)	(0.026)	(0.026)
Controls*time trend	Y	Y	Y	Y	Y
Mean of dep. variable	0.937	0.948	0.933	0.963	0.925
Observations	2368	708	1660	738	1630
R-squared	0.012	0.014	0.024	0.006	0.019
Clusters	1184	354	830	369	815

*Education Plans* is a dummy coded 1 if the respondent planned to continue in full-time education after year 11, regardless of whether they intended to take a gap year. Controls are the baseline characteristics in 2004 presented in Table 1: dummies for working status and education level of the main parent, single or cohabiting household, gender and if the individual was born in the UK. Standard errors are clustered at the individual level

***p < 0.01, **p < 0.05, *p < 0.1

No significant effect on the education plans is found for the Muslims with educated parents (those who had intermediate or higher education). This may be due to the importance of having a role model. Teenagers who do not have educated parents may not fully appreciate the importance of education and its benefits, making them more susceptible to changing their expectations of educational returns due to negative changes in their environment.

Furthermore, the analysis shows that the change in education plans was driven only by Muslims whose parents were not working. This result could be related to the increase in expectations of discrimination in the workplace for Muslims after university, as found by Hole and Ratcliffe (2020). Since unemployment is more salient for the children of parents who are not working, they may be more likely to react to negative changes in their environment, dampen their job prospects, and become uncertain about the returns of their education.^10^

**Fig. 8:**
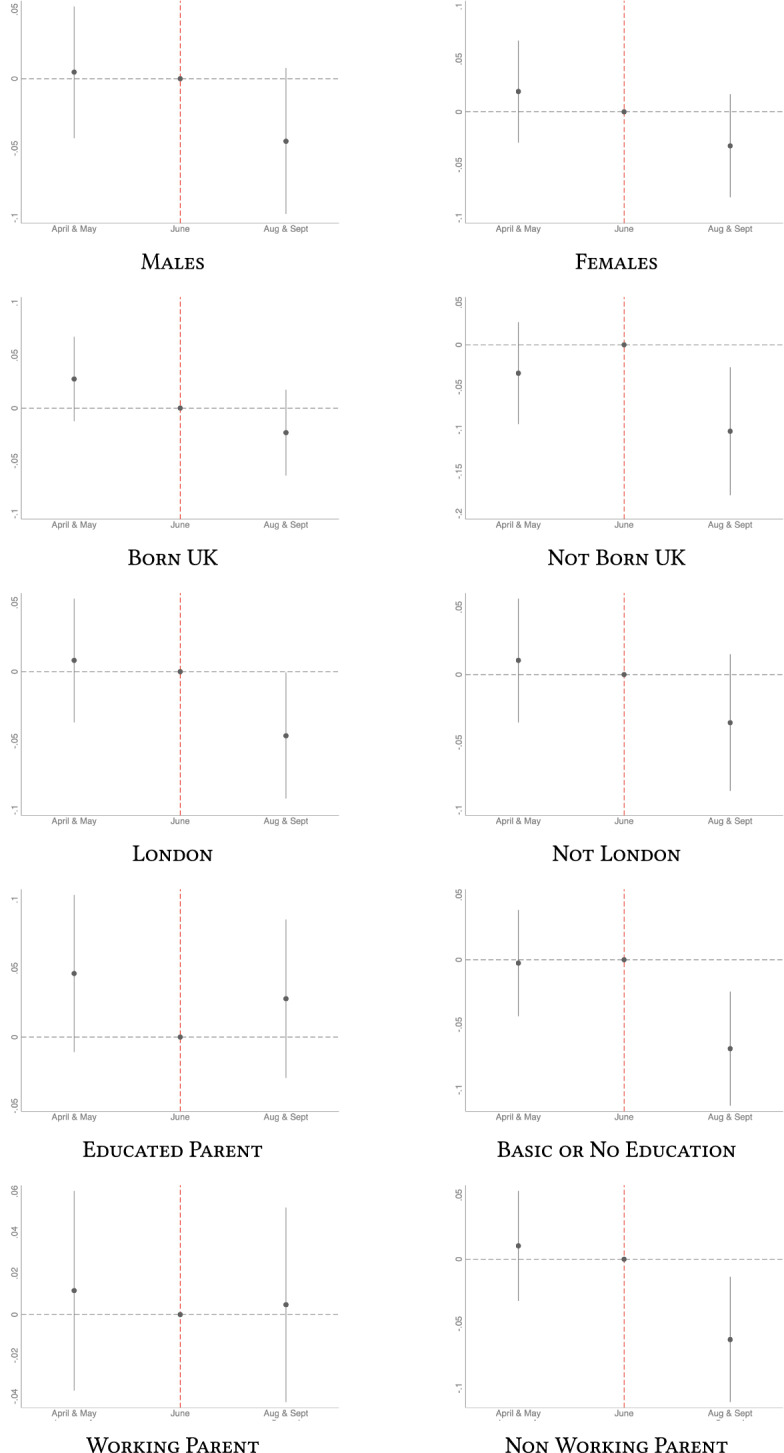
Fixed effects model by month of interview in 2005-heterogeneity

Finally, Fig. 8 presents the estimations of Eq. (3) with their 90% confidence intervals. These figures tell a similar story as no pre-trend is found and after the attack, the ones who react are those not born in the UK, those in London, and specially, those with low educated parents or parents who are not working.

## Results on education decisions

Table 7 assesses whether respondents’ answers in 2004 and 2005 affected their education decisions at age 17. For non-Muslim respondents, the order of magnitudes and signs make sense. The probability of being in education is lowest if in both 2004 and 2005 the individual stated that they were unsure or did not plan to continue full-time education. The probability starts to increase if at least in 2004 the plan was to continue in full-time education, it increases more if they had “positive” education plans in 2005, and is the highest if the education plans were positive in both years.

**Table 7:** Probit-education decisions

	Dependent variable: education decisions in 2007					
	Non-Muslims			Muslims		
	(1)	(2)	(3)	(4)	(5)	(6)
Change negative	0.253***	0.206**	0.203**	$-$0.162	$-$0.189	$-$0.145
	(0.083)	(0.084)	(0.087)	(0.381)	(0.386)	(0.388)
Change positive	0.483***	0.437***	0.427***	$-$0.000	$-$0.031	$-$0.060
	(0.083)	(0.083)	(0.084)	(0.364)	(0.372)	(0.370)
Stay positive	1.264***	1.136***	1.137***	0.642**	0.575*	0.593**
	(0.063)	(0.066)	(0.068)	(0.298)	(0.303)	(0.302)
Controls	N	Y	Y	N	Y	Y
Mean of dep. variable	0.676	0.676	0.681	0.837	0.837	0.841
Observations	6371	6371	6193	898	898	883
R-squared	0.0957	0.120	0.117	0.0278	0.0571	0.0563
Clusters	640	640	631	275	275	270

*Education Decisions* is a dummy coded 1 if the respondent’s main activity at age 17 is education. Controls are the baseline characteristics in 2004 presented in Table 1: dummies for working status and education level of the main parent, single or cohabiting household, gender and if the individual was born in the UK. Robust standard errors

***p < 0.01, **p < 0.05, *p < 0.1

The picture is less clear for the Muslim community. The only coefficient that is statistically different from 0 is $Stay\,positive$; however, the magnitude is half that of the non-Muslim community. The probability of being in education is statistically not different for those who answered $Change\,negative$, $Change\,positive$ or $Stay\,negative$. Therefore, it is very difficult to interpret the results aside from the fact that the answers from the education plans are less able to predict the actual plans for the Muslim community.

This could be interpreted as a positive sign that more than 1 year after the attacks, when the Muslim respondents had to choose whether to remain in full-time education, they readjusted their economic perspectives and put less weight on their past responses than their non-Muslim counterparts, as many of them were answering in the heat of the moment. This behavior would be in line with the results of Zorlu and Frijters (2019) as the decrease in happiness they observed was only temporarily.

The responses of the UK police and government, which condemned any reprisal against the Muslim community (Winkler 2005), might have also helped prevent the initial negative effect on education plans from translating into actual education decisions. The patterns of incidents reverted to previous levels in London 4 months after the attack Kielinger and Paterson (2013) (see “Appendix Figs. 9 and 10”).

To further study this reasoning that the responses of Muslims are just more noisy because they responded in the heat of the moment, I re-estimate Eq. (4) but adding the interaction between stated intentions between 2004 and 2005 and whether the individual is interviewed post July 2005 (treated):

5 $$\begin{aligned} \begin{array}{ll} Edu_{i}=&{}1(\alpha _0 + \alpha _{1}Change\,negative_i + \alpha _{2}Change\,positive_i \\ &{}+ \alpha _{3}Stay\,positive_i + \alpha _{4} Treated + \alpha _{5}Change\,negative_i* Treated \\ &{} + \alpha _{6}Change\,positive_i * Treated \\ &{}+ \alpha _{7}Stay\,positive_i* Treated + X_{i}'\gamma _2 + \epsilon _{i}\ge 0) \end{array} \end{aligned}$$

If the responses post-treatment for Muslims are really a temporary aberration (i.e., reflecting a temporary decline in well-being or general mood) then stated educational intentions between 2004 and 2005 should be more noisy and less correlated with actual decisions, than those responses for the Muslims that answered before the attack. In opposition, the correlation between the education plans and actual decisions should be similar for non-Muslims, independently of the timing of the interview. Table 8 present the estimation results of Eq. (5). Columns 1 and column 4 present the baseline results of Table 7 for non-Muslims and Muslims, respectively, for better comparison. Columns 2 and 4 do the same but in this case for the model with interactions. Column 3 excludes the Sikh population, given that there is suggestive evidence that the Sikh were also affected by the events after the attack.

**Table 8:** Probit-education decisions interacted with treatment

	Dependent variable: education decisions in 2007				
	Non-Muslims			Muslims	
	(1)	(2)	(3)	(4)	(5)
Change negative ($\alpha _1$)	0.206**	0.204**	0.188**	$-$0.189	0.001
	(0.084)	(0.093)	(0.093)	(0.386)	(0.469)
Change positive ($\alpha _2$)	0.437***	0.476***	0.463***	$-$0.031	0.152
	(0.083)	(0.093)	(0.093)	(0.372)	(0.424)
Stay positive ($\alpha _3$)	1.136***	1.135***	1.110***	0.575*	0.593*
	(0.066)	(0.073)	(0.073)	(0.303)	(0.349)
Treated ($\alpha _4$)		0.081	0.018		0.317
		n (0.150)	(0.151)		(0.749)
Change negative*Treated ($\alpha _5$)		0.014	0.017		$-$0.640
		(0.206)	(0.211)		(0.828)
Change positive*Treated ($\alpha _6$)		$-$0.225	$-$0.139		$-$0.834
		(0.208)	(0.214)		(0.871)
Stay positive*Treated ($\alpha _7$)		0.004	0.052		$-$0.155
		(0.159)	(0.160)		(0.772)
Controls	Y	Y	Y	Y	Y
Sum of coefficients					
$\alpha _1 + \alpha _5$=		0.218	0.205		$-$0.639
$\alpha _2 + \alpha _6$=		0.251	0.323*		$-$0.682
$\alpha _3 + \alpha _7$=		1.139***	1.162***		0.438
Mean of dep. variable	0.676	0.676	0.676	0.837	0.837
Observations	6371	6371	6169	898	898
R-squared	0.120	0.120	0.122	0.0571	0.0611

*Education Decisions* is a dummy coded 1 if the respondent’s main activity at age 17 is education. Controls are the baseline characteristics in 2004 presented in Table 1: dummies for working status and education level of the main parent, single or cohabiting household, gender and if the individual was born in the UK. Column 3 excludes the Sikh population, given that they seemed also affected by the attack. Robust standard errors

*** p < 0.01, ** p < 0.05, * p < 0.1

For the non-Muslims, qualitatively, the estimated parameters appear not to change radically between the treatment (stated intention + interaction) and the control group (stated intention), as they are very similar. The direction of the estimations is still in line to what logic would dictate, as is more probable to continue studying if at least in 2004 the plan was to continue in full-time education, comparing it to someone who both years stated their insecurity about continuing studying full-time in the future. This probability increases more if they had a “positive” change on education plans in 2005, and is the highest if the education plans were positive in both years. In terms of significance, although the coefficient of $Change\,negative_i$ stops being significant for the treated, the other two coefficients in Column 3 $Change\,positive_i$ and $Stay\,positive_i$ remain significant for both treated and control. This suggests that for the treated non-Muslims, the stated education plans still have predictive power on the actual education decisions.

For the Muslims, before comparing between treatment and control group, it is worth noticing that even before the attack, the stated education plans are less in line with the education decisions for the Muslims than for the non-Muslim, which is surprising. Previous to the attack, in the case of the Muslims, the only coefficient that is statistically different from 0 is $Stay\,positive$, in clear contrast with the control group for the non-Muslims. Nevertheless, the difference between the responses of Muslims before and after the attack is much more different than for the non-Muslims. Qualitatively, the coefficients make less sense at the time of interpreting them after the attacks. Not only that, but, all the coefficients for the treated Muslims, including $Stay\,positive$, become insignificant, therefore, the stated education plans for the Muslims interviewed after the terrorist attack are no good in predicting the education decisions of these individuals, not even if the student responded positively twice about her intention of continuing in full-time education.

**Fig. 11:**
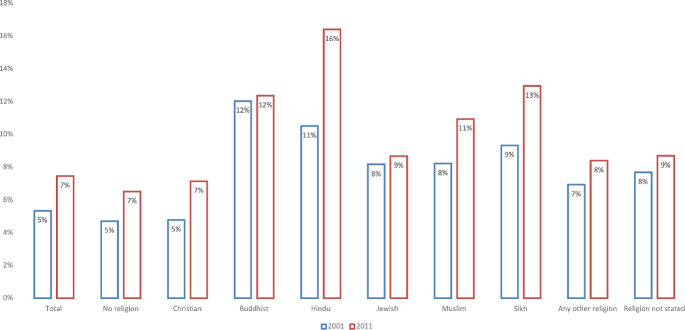
Percentage of persons age 19–21 with qualification of level 4 or more in the UK. *Note*: The Office for National Statistics defines level 4 and above as: Degree (for example BA, BSc), Higher Degree (for example MA, PhD, PGCE), NVQ Level 4–5, HNC, HND, RSA Higher Diploma, BTEC Higher level, Foundation degree (NI), Professional qualifications (for example teaching, nursing, accountancy)

Furthermore, using census data from the UK Official National Statistics, I discovered that between the 2001 and 2011 censuses, the percentage of individuals aged 19–21 in the Muslim community who obtained a Level 4 or higher qualification (typically acquired through full-time education) increased. Muslims experienced some of the largest gains among religious groups, as shown in Fig. 11 in the “Appendix.” As this age group were teenagers in 2005, if the uncertainty expressed by Muslim teenagers about their education plans after the terrorist attack had persisted, one would not expect to see this trend in qualifications among Muslims. Taken together with the results from the previous tables, this pattern suggests that any change in plans to remain in full-time education as a result of the terrorist attack may have been temporary. However, this is merely suggestive evidence, as it is possible that this trend could have been even more pronounced in the absence of the attack, or it could be due to migration patterns and positive self-selection of Muslims who chose to stay in the country.

Finally, it is important to consider the dropout rates between surveys. “Appendix Table 17” indicates that there may be a higher attrition rate for Muslims who intended to stay in full-time education by the age of 16 compared to non-Muslims with the same plans. This may contribute to the apparent unpredictability of education decisions among Muslims interviewed after the attack, as small sample sizes could impact statistical precision. It is worth noting, however, that the difference in attrition rates between Muslims and non-Muslims becomes insignificant when baseline controls are added.

More research is needed in this area. Although it seems the terrorist attack did not affect whether the Muslim students surveyed remained in full-time education in the extensive margin, it might have influenced their education and career paths. Carlana et al. (2022), for example, find that on average, migrants in Italy are more likely to enroll in vocational high schools than natives of similar ability, who choose more technical or academic-oriented high schools. They find that tutoring and career counseling can increase the number of migrants enrolling in high-skills-track high schools. One potential avenue for further research would be to study the education choices of Muslim teenagers after the terrorist attack. The Next Steps survey data can be merged with the National Pupil Database via request to the UK Department for Education to explore the respondents’ grades and educational achievements as well as their subject choices and the type of education they pursued. Another option could be to use the Youth Questionnaire (for 11-15 year-olds) from the British Household Panel Survey (BHPS). This questionnaire includes multiple questions about teenagers’ education, training and aspirations and provides not only the month of the interview but also the date. These findings would deepen the analysis of whether the attack affected the *actual* educations of the Muslim students aside from their education plans.

## Conclusion

These study’s results found that the 7/7 attack in London negatively affected young Muslims’ educational *plans*, but not those of respondents from other religious groups. The effect of changes in expectations does not appear to be driven by the announcement that the Olympic Games would be held in London in 2012, or by any other individual characteristic. These changes in education plans results seem to be driven by Muslims that experienced a worst backslash against their community and that have a potential lower integration with their host economy.

However, the evidence is inconclusive as to whether these plans affected the Muslim respondents’ actual educational outcomes. A potential explanation is that in the heat of the moment, Muslim teenagers reacted more negatively toward education after the attack but later reconsidered. This could be due to the efforts of the UK police and government to prevent violence and prejudice toward Muslims because of the terror acts,^11^ or because the economic payoffs of studying more provided sufficient incentives to overcome the effect of the attack, as explained theoretically by Becker and Rubinstein (2011). The evidence suggests that this might indeed be the case, as responses about education plans have less power to predict actual education decisions for Muslims than for non-Muslims. Furthermore, official national statistics demonstrate that the percentage of Muslims aged 19–21 achieving level 4 or higher qualifications increased from 2001 to 2011 even more than other religions. However, Muslim respondents’ higher attrition rate from the survey makes it difficult to arrive at a definitive conclusion.

Future research should examine whether the terrorist attack affected the intensive margin of education decisions, the course taken by the different groups, their grades or career paths. It is also important to understand what determines the persistence of education plans on education decisions, and whether the reaction by the police and government in penalizing violence against the Muslim community helped mitigate a potential negative effect in education outcomes.

## Figures and Tables

**Fig. 9:**
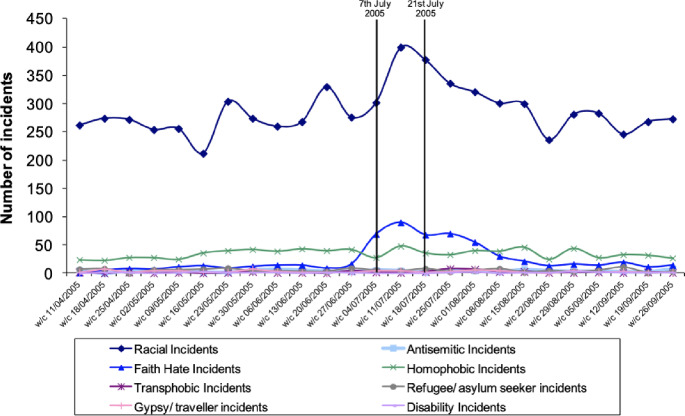
Types of hate crime incidents (April 11–October 2, 2005)

**Fig. 10:**
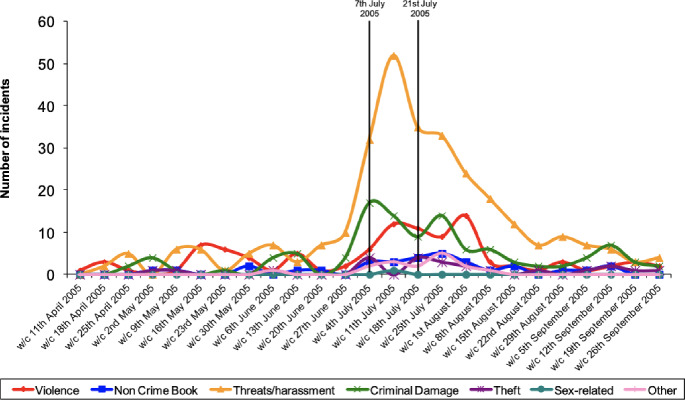
Faith incidents—allegation grouping (April 11–October 2, 2005)

## Appendix

### Descriptive statistics-next steps

**Table 9:** Descriptive statistics-main sample

	Muslim	Non-Muslim	Difference
*A. General information*			
Education plans	0.938	0.824	0.114***
Born in the UK	0.781	0.951	$-$0.170***
*B. Working status-main parent*			
Working Full Time	0.220	0.412	$-$0.192***
Working Part Time	0.092	0.342	$-$0.250***
*C. Education level-main parent*			
No qualifications	0.645	0.169	0.476***
Basic	0.056	0.115	$-$0.059***
Intermediate	0.188	0.450	$-$0.263***
Advance	0.112	0.266	$-$0.154***
*D. Household type*			
Single parent	0.208	0.253	$-$0.045***
Married or cohabiting	0.792	0.747	0.045***
Observations	1184	7932	

*Notes*: *Education Plans* is a dummy indicating whether the teenager plans to stay in full-time education (either continously or after a gap year)

****p* < 0.01, ***p* < 0.05, **p* < 0.1

### Additional tables and analysis

#### A more flexible outcome variable of education plans

One caveat in the article is the definition of education plans done in the paper. To be able to treat it as a dummy, as explained in the main text, I code the answers “don’t know” and “leave full-time education” as 0 for the variable *Education Plans*. I assign a value of 1 if the respondent said she was returning to full-time education or planning to take a gap year before returning to full-time education. However, it is true that by doing so, I am combining different responses in which one is definitely more negative toward future education plans than the other. It is not the same for an individual to state after the attack that she is unsure about continuing in full-time education than to say that she does not plan to continue in full-time education.

Table 10 allows to clarify whether the negative reaction toward education plans after the attack seen in the main results are driven by more uncertainty in the future (don’t know) or by a negative education plan all together, or both.

**Table 10:** Education plans by Muslims in 2004 and 2005 by treatment and control

	Control Muslims		Treated Muslims	
	2004 (%)	2005 (%)	2004 (%)	2005 (%)
Stay on in full-time education	92.3	93.7	95.6	92.1
Leave full-time education	2.7	2.6	2.4	2.7
Don’t know	4.2	3.3	2.1	5.0
Gap year	0.8	0.4	0.0	0.3

Distribution of Muslims in the main analysis

The decrease in the percentage of Muslims in the treated group that planned to continue in full-time education is due to the increase in the group that felt uncertain about whether they would continue in full-time education or not. Notice that the percentages in the control group remain very similar over the years, which is the initial idea of the difference-in-difference methodology. This suggests that a negative reaction in education plans in the main analysis should be regarded as Muslim teenagers becoming more uncertain about their education plans, rather than more negative.

To further study if it is in fact Muslims becoming more uncertain, instead of treating education plans as a dummy, I treat it as a categorical variable and estimate a multilogit, using as a baseline the category of continue in full-time education. Table 11 presents the result of those estimations.

**Table 11:** Using a more flexible measure of education plans

	Multilogit (baseline scenario: continue in full-time education)				
Dependent variable	Don’t know	Leave full-time education	Gap year	Education plans	Education plans
	(1)	(2)	(3)	(4)	(5)
Treated	$-$0.762*	$-$0.185	$-$15.811***		
	(0.421)	(0.424)	(0.389)		
$Year_{2005}$	$-$0.241	$-$0.063	$-$0.866	0.028	0.015
	(0.219)	(0.272)	(0.696)	(0.026)	(0.021)
Treated*$Year_{2005}$	1.173**	0.227	15.715***	$-$0.043***	$-$0.004
	(0.477)	(0.517)	(1.222)	(0.016)	(0.013)
Observations	2368	2368	2368	2258	2218
R2	0.050	0.050	0.050	0.012	0.005
Mean	0.937	0.937	0.937	0.964	0.974
Clusters	1184	1184	1184	1129	1109

*Education Plans* is a dummy coded 1 if the respondent planned to continue in full-time education after year 11, regardless of whether they intended to take a gap year. In column 4, it takes the value of 0, only if the respondent answered “Don’t know” and in column 5, it takes the value of 0 only if the respondent planned to leave full-time education. Controls are the baseline characteristics in 2004 presented in Table 1: dummies for working status and education level of the main parent, single or cohabiting household, gender and if the individual was born in the UK. Standard errors are clustered at the individual level

****p* < 0.01, ***p* < 0.05, **p* < 0.1

Focusing on the estimations of the multilogit, Muslim teenagers surveyed after the attack were more likely to express uncertainty about their education plans rather than to express their plans of continue studying full time, as suggested by the coefficient of interaction. This is not the case for the category of leaving full time education where there is no significant difference between being interviewed before or after the attack. Both results are consistent with Table 10. Notice that after the attack, it is also more likely for Muslims teenagers to respond their intention to take a gap year that continuing right away in full-time education, but since it is not clear whether this is negative or not toward capital accumulation, this result is left out of the analysis.

Column 4 of Table 11 estimates the main fixed effect model (Eq. 1) but excluding the Muslims that planned to leave full-time education. Column 5 excludes those who were not sure whether to continue studying or not full-time. Again, it seems the results are driven exclusively by the changes toward the category “Don’t know.” Because of this result, the interpretation should be made that the attack, rather than making the plans of continue in full-time education more negative, it made them more uncertain.

#### Using month of interview in 2005 instead of treatment and control

See Table 12.

**Table 12:** Fixed effects model by month of interview 2005

	Dependent variable: education plans		
	(1)	(2)	(3)
$Year_2005$	0.005	0.035	0.035
	(0.015)	(0.033)	(0.034)
May*$Year_{2005}$	0.009	0.011	0.013
	(0.020)	(0.020)	(0.021)
August*$Year_{2005}$	$-$0.037*	$-$0.039*	$-$0.043*
	(0.021)	(0.022)	(0.022)
Controls*time tend	N	Y	Y
Mean of dep. variable	0.937	0.937	0.937
Observations	2368	2368	2316
R2	0.004	0.012	0.012
Clusters	1184	1184	1158

*Education Plans* is a dummy coded 1 if the respondent planned to continue in full-time education after year 11, regardless of whether they intended to take a gap year. Controls are the baseline characteristics in 2004 presented in Table 1: dummies for working status and education level of the main parent, single or cohabiting household, gender and if the individual was born in the UK. Standard errors are clustered at the individual level

****p* < 0.01, ***p* < 0.05, **p* < 0.1

#### Main effects: including those interviewed on July in the sample

See Table 13.

**Table 13:** Effects of the terrorist attack on education plans

	Dependent variable: education plans						
	FE			LPM		Probit	
	(1)	(2)	(3)	(4)	(5)	(6)	(7)
Treated				0.019	0.023*	0.155	0.188*
				(0.012)	(0.012)	(0.100)	(0.102)
$Year_{2005}$	0.009	0.028	0.029	0.009	0.011	0.078	0.089
	(0.010)	(0.027)	(0.028)	(0.010)	(0.010)	(0.082)	(0.082)
Treated*$Year_{2005}$	$-$0.024*	$-$0.025*	$-$0.029**	$-$0.024*	$-$0.028**	$-$0.202*	$-$0.240**
	(0.014)	(0.014)	(0.015)	(0.014)	(0.014)	(0.121)	(0.122)
Controls	N	N	N	Y	Y	Y	Y
Controls*time trend	N	Y	Y	N	N	N	N
Average marginal effects							
						$-$0.023*	$-$0.027**
						(0.014)	(0.014)
Mean of dep. variable	0.939	0.939	0.939	0.939	0.939	0.939	0.939
Observations	3318	3318	3244	3318	3244	3318	3244
R-squared	0.002	0.007	0.007	0.011	0.012	0.026	0.027
Clusters	1659	1659	1622	1659	1622	1659	1622

*Education Plans* is a dummy coded 1 if the respondent planned to continue in full-time education by year 11, regardless of whether they intended to take a gap year. Columns 3. 5 and 7 restrict the sample to non-movers. Controls are baseline characteristics in 2004 presented in Table 1: dummies for working status and education level of the main parent, single or cohabiting household, gender and if the individual was born in the UK. Standard errors are clustered at the individual level

****p* < 0.01, ***p* < 0.05, **p* < 0.1

#### Differential attrition rate of the Muslims after the attack

Ideally, to study the differential attrition of Muslims before and after the attack, I would need to know the month of interview in 2005 of the Muslims who decided not to continue with the survey and check whether attrition increased in 2005 after the attack. However, this is not possible, as these teenagers did not respond in 2005. What can be done is to use information from the survey in 2004 to study whether this attrition rate is a problem in the analysis. Table 14 shows the distribution by month of the Muslims used in the main analysis, divided by treated and non-treated.

**Table 14:** Distribution of Muslims by month of interview in 2004

Month of interview	Non-treated Muslims	%	Treated Muslims	%
April	220	26.1	33	9.7
May	216	25.6	63	18.5
June	201	23.8	79	23.2
July	138	16.4	102	30.0
August	69	8.2	63	18.5
Total	844	100	340	100

Distribution of Muslims in the main analysis by month of interview in 2004

Most of the treated Muslims were interviewed in 2004 in the months of June, July and August; meanwhile, most of the non-treated Muslims were interviewed in April, May and June. Therefore, if the probability of dropping out of the survey in 2005 increases for the groups that were interviewed in the months of August or July in 2004, it would be a problem as it is more likely that those who dropped out were to be treated had they answered the 2005 survey. Table 15 estimates the probability of dropping out of the survey in 2005 depending on the month of interview in 2004. No significant differences in this probability were found, suggesting that a differential attrition rate after the attack, although it is impossible to check directly, is of little concern in the analysis.

**Table 15:** Probability of Muslims dropping out of the 2005 survey

	Dependent variable: drop out in 2005		
	LPM	LPM	Probit
May$_{2004}$	$-$0.022	$-$0.025	$-$0.085
	(0.033)	(0.033)	(0.104)
June$_{2004}$	0.023	0.021	0.063
	(0.032)	(0.032)	(0.101)
July$_{2004}$	$-$0.041	$-$0.043	$-$0.144
	(0.034)	(0.034)	(0.110)
August$_{2004}$	0.017	0.013	0.035
	(0.040)	(0.040)	(0.126)
Controls	N	Y	Y
Mean of dep. variable	0.248	0.248	0.248
Observations	1575	1575	1575
R-squared	0.003	0.014	0.012

*Drop out in 2005* is a dummy coded 1 if the Muslim respondent dropout from the survey in 2005. Dummies of month of interview in 2004 are included. Additional controls are the baseline characteristics in 2004 presented in Table 1. Standard errors are clustered at the individual level

****p* < 0.01, ***p* < 0.05, **p* < 0.1

#### Heterogeneity analysis by month of interview in 2005

See Fig. 8.

#### Mechanism as proposed by Hole and Ratcliffe (2020)

Unfortunately, it is not possible to estimate Eq. (1) with the new dependent variable since the questions regarding happiness overall and job market expectations were only included in the 2005 survey. As an alternative, I follow the approach of Hole and Ratcliffe (2020) and use a differences-in-differences model to compare changes in outcomes between Muslims and other religions, who serve as a control group, before and after the attack.

6 $$\begin{aligned} Y_{i}&= \alpha _0+ \alpha _{1}Post_i + \alpha _{2}Muslim_i + {\alpha _{3}}Post_i*Muslim_i + X_{i,2004}'*\gamma + u_{i} \end{aligned}$$

In Eq. (6), *Post* is a dummy coded 1 if the month of interview in 2005 is after June and *Muslim* is a dummy coded 1 if the respondent was Muslim. The coefficient of interest would be the interaction between *Muslim* and *Post*. As dependent variables, I create dummies for all possible answer to the question *“Have you recently been feeling reasonably happy, all things considered?”*, being those answers: “More so than usual,” “About the same as usual,” “Less so than usual” and “Much less than usual.” I excluded the ones that answer “Don’t know” as it is harder to interpret. I also construct a dummy coded 1 if the respondent answer positively to the question *“Do you think that your skin color, ethnic origin or religion will make it more difficult for you to get a job after you leave education?”*. Results are presented in Table 16. Results are similar to the ones found in the original paper although some of the controls are different,^12^

**Table 16:** Linear probability models

Dependent variable	Much less than usual	Less so than usual	About the same as usual	More so than usual	As usual or more	Discrimination
	(1)	(2)	(3)	(4)	(5)	(6)
Post	0.000	$-$0.005	$-$0.007	0.011	0.005	0.016***
	(0.004)	(0.006)	(0.010)	(0.009)	(0.007)	(0.005)
Muslim	$-$0.015**	$-$0.016	$-$0.037*	0.067***	0.030**	0.084***
	(0.007)	(0.011)	(0.020)	(0.019)	(0.012)	(0.016)
Muslim*Post	0.011	0.029*	$-$0.015	$-$0.025	$-$0.040**	0.054**
	(0.010)	(0.015)	(0.028)	(0.026)	(0.017)	(0.023)
Controls	Y	Y	Y	Y	Y	Y
Mean of dep. variable	0.0320	0.0812	0.611	0.276	0.887	0.0673
Observations	11,102	11,102	11,102	11,102	11,102	9983
R2	0.007	0.010	0.004	0.012	0.017	0.031

Dependent variables from columns 1 to 4 are a dummy coded 1 if the answer by the respondent to the question “Have you recently: been feeling reasonably happy, all things considered?” was the one written in the column. Column 5 is a dummy coded 1 if the answer to the former question was either as happy as usual or more happy than usual. *Discrimination* is a dummy coded 1 if the respondent was expecting difficulties in the job market after education, because of its color of skin or religion. *Post* is a dummy coded 1 if the month of interview in 2005 is after June and *Muslim* is a dummy coded 1 if the respondent was Muslim. Additional controls are the baseline characteristics in 2004 presented in Table 1. Robust standard errors

****p* < 0.01, ***p* < 0.05, **p* < 0.1

Table 16 indicates that following the terrorist attacks, Muslims were more likely to report feeling less happy than usual, as shown in columns 2 and 5. Additionally, they were more likely to anticipate discrimination in the job market based on their skin color, ethnicity, or religion.

Establishing causality in this analysis is more challenging than in the main results of the article because it depends on the assumption that other religions had similar pretrends to the Muslim community. Although these results are not new, they provide suggestive evidence of how a terrorist attack could affect the education plans of the Muslims.

#### Attrition rate in 2007

Around 20% of the non-Muslim teenagers are not present in the wave 4, when education decisions are being recorded.

**Table 17:** Survey attrition by 2007

Change in education plans	Muslim	Non-Muslim	Difference
Stay Negative	0.250	0.285	$-$0.035
Change Negative	0.365	0.272	0.094
Change Positive	0.265	0.253	0.013
Stay Positive	0.234	0.172	0.062***

Mean of Muslim and non-Muslim teenagers out of the survey by 2007. The last column is the difference between the mean of both groups

****p* < 0.01, ***p* < 0.05, **p* < 0.1

Table 17 presents the proportion of individual Muslim and non-Muslim respondents that left the survey according to their responses on education plans. It also shows the difference in means between both groups in each category. The attrition rate among Muslims is higher in the group that stayed positive in continuing full-time education in both years. A probit on the probability of being out of the survey on the characteristics reveals that respondents were less likely to drop out of the survey if they were born in the UK and if their parents were more educated and working part time or full time. Since these characteristics are associated with a higher probability of being in full-time education for Muslim teenagers (with the exception of being born in the UK), then the drop out rate is important. If those who are dropping out of the survey are the ones not staying in full-time education, then the coefficients for the Muslims in Table 7 can be biased downwards as the pool of stayers in the Muslim group is more selected than in the non-Muslim group. It is worth mentioning that the difference in attrition rates between Muslims and non-Muslims stop being significant when adding baseline controls.

## Footnotes

1 See, for example, BBC News, “Hate crimes soar after bombings,” August 4, 2005. http://news.bbc.co.uk/2/hi/uk_news/england/london/4740015.stm; BBC News, “Race body reports rise in abuse,” July 22, 2005. http://news.bbc.co.uk/1/hi/wales/south_east/4704593.stm.

2 See, for example, BBC News, “Sikh meet police after the attacks,” July 12, 2005. http://news.bbc.co.uk/2/hi/uk_news/4674883.stm.

3 For a general review of the effect of violence on different outcomes, see Verwimp et al. (2019).

4 See: https://nextstepsstudy.org.uk/.

5 The results are somewhat stronger if the gap year is counted as 0, but the interpretation is less clear.

6 The results do not change if instead of main parent I use information only about the mother (there is missing information about the father in nearly a quarter of the sample).

7 The reason for restricting the sample like this is twofold. Firstly, it allows to rule out migration as a possible reason for the negative reaction to education plans. For example, if Muslims after the attack moved between regions more than Muslims before the attack, it could indicate that the negative reaction is due to them not liking their new region as much. Secondly, even between Muslims that stayed in the same region, the terrorist attack might affect the decision to move to another school. For example, after the attack, Muslim parents might choose to reallocate their daughter or son to a school they believe is more tolerant to avoid the possibility of bullying. By restricting the sample to those who did not move to another school, it ensures that any negative reaction in education plans is not due to the teenagers disliking their new school for some unrelated reason.

8 BBC News, “Sikh meet police after the attacks,” July 12, 2005. http://news.bbc.co.uk/2/hi/uk_news/4674883.stm.

9 The changes in education plans, although high relative to the baseline, are only around 4 percentage points. This is because most teenagers plan to continue in full-time education. Hence, to ensure accuracy, it is important to properly classify who was interviewed before and after the attack, due to the risk of miss-classifying 1/4 of the ones interviewed in July (assuming a constant distribution of interviews within the month). This could add too much noise in the data.

10 It is important to keep in mind that, while it may be that a decline in mental health causes poor educational outcomes, it could just be that general mood (i.e., feelings of pessimism/despair evoked by the social environment after the bombings) affects the way that Muslims respond to all attitudinal questions, including those related to mental health and educational intentions. The information used in the paper does not allow us to differentiate between the two possibilities. However, it is unlikely that this is the only explanation for the results, as the effect on education plans is more pronounced among those with less educated/financially secure parents, who may have less capacity to protect their children against changes in circumstances. If it were just general mood, one might expect it to affect everyone’s responses in the same way.

11 BBC News, “Police vows over revenge attacks,” July 12, 2005. http://news.bbc.co.uk/2/hi/uk_news/4674079.stm.
